# Decoding RNA–Protein Interactions: Methodological Advances and Emerging Challenges

**DOI:** 10.1002/ggn2.202500011

**Published:** 2025-05-12

**Authors:** Wenkai Yi, Jian Yan

**Affiliations:** ^1^ Department of Biomedical Sciences Tung Biomedical Sciences Centre City University of Hong Kong Kowloon Tong Hong Kong; ^2^ Department of Precision Diagnostic and Therapeutic Technology The City University of Hong Kong Shenzhen Research Institute Shenzhen 518016 China; ^3^ Department of Urology The First Affiliated Hospital of Xi'an Jiaotong University Xi'an 710061 China

**Keywords:** Protein‐centric methods, RNA, RNA‐binding proteins, RNA‐centric methods, RNA–protein interactions

## Abstract

RNA–protein interactions are fundamental to cellular processes such as gene regulation and RNA metabolism. Over the past decade, significant advancements in methodologies have transformed the ability to study these interactions with unprecedented resolution and specificity. This review systematically compares RNA‐ and protein‐centric approaches, highlighting their strengths, limitations, and optimal applications. RNA‐centric methods, including hybridization‐based pulldowns, proximity labeling, and CRISPR‐assisted techniques, enable the identification of proteins interacting with specific RNAs, even low‐abundance or transient partners. Protein‐centric strategies, such as immunoprecipitation‐based CLIP‐seq, and emerging proximity‐tagging methods, map RNA interactomes of RNA‐binding proteins with nucleotide precision. This study evaluates key innovations like LACE‐seq and ARTR‐seq, which minimize cell input requirements, and HyPro‐MS, which bypasses genetic modifications. Guidelines for method selection are provided, emphasizing experimental goals, RNA abundance, interaction dynamics, and technical constraints. Critical challenges are also discussed, including capturing low‐affinity interactions, resolving RNA structural complexities, and integrating multi‐omics data. This review underscores the importance of method‐tailoring to biological contexts, offering a roadmap for researchers to navigate the evolving landscape of RNA–protein interaction studies. By bridging technical advancements with practical recommendations, this study aims to accelerate discoveries in RNA biology, therapeutic development, and precision medicine.

## Graphical abstract

This review provides a comprehensive comparison of RNA‐ and protein‐centric methodologies for investigating RNA–protein interactions, emphasizing their respective advantages, limitations, and suitable applications. It highlights recent technological innovations, outlines strategic method selection criteria, and addresses key challenges. By integrating technical insights with practical guidance, the review supports advances in RNA biology, therapeutic development, and precision medicine.

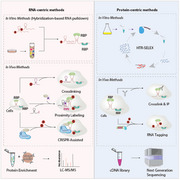

## 1 Introduction

More than 70% of the human genome can be transcribed into RNA during developmental stages, most of which are noncoding RNAs (ncRNAs).^[^ ^1^ ^]^ An increasing body of evidence suggests that RNAs always work in tandem with RNA‐binding proteins (RBPs) to form ribonucleoprotein complexes (RNPs).^[^ ^2^, ^3^ ^]^ RNA–protein interactions are fundamental to the survival and functionality of cells, as these interactions play critical roles in regulating various cellular processes, including transcription, splicing, translation, and mRNA decay, directly impacting cell behavior and influencing the development and progression of various diseases.^[^ ^4^, ^5^ ^]^ The interacting proteins dictate the function of RNA. For example, the X‐inactive specific transcript (XIST), one of the first identified long noncoding RNAs (lncRNAs) is essential for X‐chromosome inactivation (XCI) in female mammals and key for dosage compensation between the sexes.^[^ ^6^, ^7^ ^]^ Various technologies have revealed proteins binding to XIST,^[^ ^8^, ^9^, ^10^, ^11^, ^12^, ^13^ ^]^ including SPEN, hnRNPK, hnRNPU, and BRG1. SPEN has been found to serve as a scaffold to recruit transcriptional repressor complexes, ensuring the X chromosome remains inactive for dosage compensation.^[^ ^14^, ^15^ ^]^ Similarly, hnRNPK can recruit chromatin‐modifying complexes to facilitate transcriptional silencing.^[^ ^16^ ^]^ Both proteins promote phase separation with XIST, creating a transcriptionally repressive environment on the X chromosome.^[^ ^17^, ^18^ ^]^ The results unravel XIST's biological role in XCI, underscoring the value of identifying RNA‐binding proteins in understanding the function of lncRNA.

These interactions between RNAs and RBPs are a highly intricate process that is meticulously regulated and orchestrated within the cellular environment, with RNA structure and RNA‐binding domain (RBD) playing a pivotal role in these interactions.^[^ ^19^, ^20^ ^]^ The structural attributes of RNA, including double‐stranded regions and various secondary structures such as hairpins, loops, and stems, are critical determinants of its binding specificity and affinity to proteins.^[^ ^21^ ^]^ RBDs, such as RNA recognition motif (RRM), K homology (KH), and zinc finger (ZNF), within proteins are the functional units responsible for RNA binding, and the presence of multiple such domains within a single RBP enables coordinated and enhanced binding to RNAs.^[^ ^22^ ^]^ The study of RNA–protein interactions presents challenges due to their complexity and dynamic nature.^[^ ^23^ ^]^ These interactions can vary in terms of stability and strength, ranging from transient to stable and from weak to strong. In addition, both RNAs and RBPs can undergo various modifications that can influence their interactions.^[^ ^20^ ^]^

The study of RNA–protein interactions demands a diverse toolkit of experimental strategies, each tailored to capture the dynamic and context‐dependent nature of these molecular partnerships. Over the past several decades, methodologies for RNA–protein interactions detection have evolved from traditional in vitro binding assays to hybridization‐based strategies, culminating in the development of sophisticated in vivo proximity‐labeling methods. Indeed, the advancements in methodologies for detecting RNA–protein interactions have provided researchers with increasingly detailed resolution, both spatially and temporally. These developments have not only highlighted the inherent complexity of RNA–protein interactions, but also underscored the necessity for precise, contextually relevant data in elucidating their regulatory roles in binding processes. Broadly, the methodologies for detecting RNA–protein interactions fall into two widely recognized categories: RNA‐centric methods and protein‐centric methods.^[^ ^24^, ^25^ ^]^ RNA‐centric methods, which initiate with a specific RNA molecule to identify its protein interactors, are incredibly effective in revealing proteins associated with distinct transcripts, including noncoding RNAs or viral genomes. RNA‐centric approaches confer significant insights into the protein interactome of distinct RNAs. However, they often require substantial initial material to ensure the isolation of a sufficient protein quantity for detection through mass spectrometry. This requirement presents potential difficulties, particularly when the RNA of interest is of low abundance, or when the initial material is limited, as is often the case with clinical samples or rare cellular populations. Conversely, protein‐centric methods, which begin with a specific RBP to map its RNA targets, are indispensable for decoding the RNA interactomes of target RBP. They typically require less starting material compared to RNA‐centric methods, thereby offering a practical solution to investigating samples that are limited or of high value. Notably, the sensitivity of certain protein‐centric techniques can extend to the single‐cell level or single‐nucleotide resolution. In the following sections, we will extensively examine the principles, comparative advantages and disadvantages, and applications of current RNA–protein interactions detection methods, setting the stage for a critical evaluation of their roles in advancing our understanding of the RNA–protein interactome.

In this review, we aim to provide a comprehensive overview of the most recent methodologies used for detecting RNA–protein interactions and to discuss their relevance and application across different types of RNA molecules. While we have highlighted key advancements and widely adopted methods, it is important to note that this review will not encompass every available technique. By understanding these methodologies, researchers can better apply them to elucidate the roles of RNA–protein interactions in cellular processes and disease mechanisms, ultimately contributing to the development of novel therapeutic strategies.

## 2 Methods for RNA–Protein Interactions Detection

### 2.1 RNA‐Centric Methods

**Figure 1:**
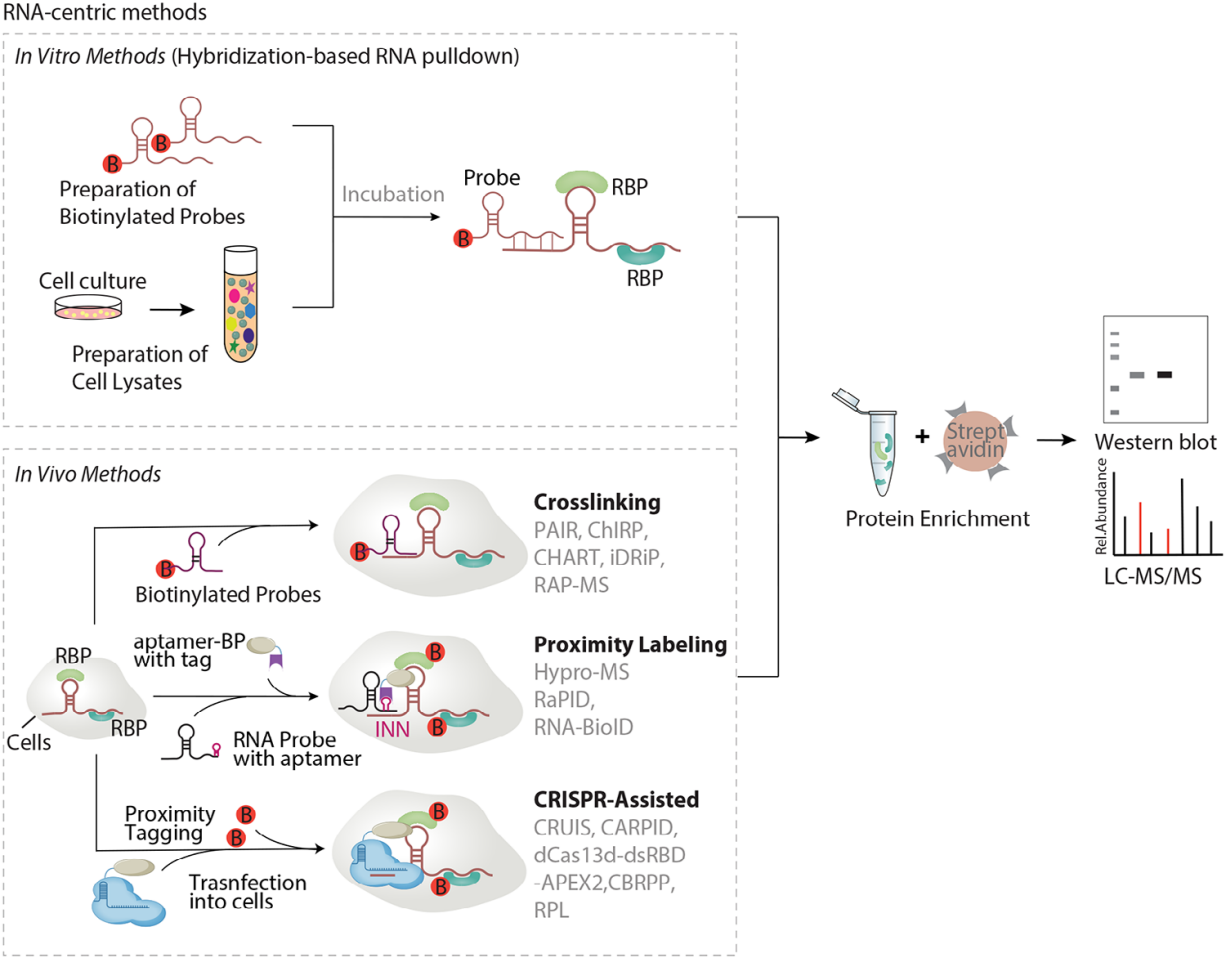
Schematic overview of RNA‐centric methods. in vitro methods (upper): RNA is synthesized with a biotin or aptamer tag at one end and incubated with cellular protein extracts, allowing the binding of RNA to interacting proteins. in vivo methods (lower): Hybridization‐based RNA pulldown techniques involves crosslinking cells using formaldehyde, glutaraldehyde, or UV light. Biotinylated oligonucleotide probes are then hybridized to the RNA of interest. In proximity labeling–based approaches, enzymes such as biotin ligase or peroxidase label proteins in close proximity to the target RNA, which is typically tagged with aptamers (e.g., MS2 or BoxB stem loops). CRISPR–assisted proximity labeling methods utilize catalytically inactive CRISPR/Cas13 protein fused to labeling enzymes to target endogenous RNAs at specific sites. Finally, biotinylated RNA‐binding proteins are enriched using streptavidin beads and identified by mass spectrometry (MS) or detected with western blot (WB).

RNA‐centric methods focus on identifying proteins that interact with a specific RNA molecule, offering insights into the dynamic roles of RNAs in cellular processes such as gene regulation, localization, and stability. These approaches are broadly categorized into *​in vitro* and *​in vivo* strategies, each tailored to address distinct biological questions (**Figure** **1**). in vitro methods, such as ​biotinylated RNA pulldown and ​aptamer‐based capture (e.g., S1 or Cys4 adaptors), use synthetic RNA of interest incubated with cell lysates to isolate bound proteins, enabling precise mapping of RNA–protein binding sites. However, they may lack physiological relevance due to the absence of native RNA modifications or cellular context. In contrast, in vivo methods use crosslinking agents like ​formaldehyde or ​Ultraviolet (UV) light to stabilize transient RNA–protein interactions within living cells, followed by RNA–protein complex purification using techniques such as RNA Antisense Purification (​RAP), ​or Chromatin Isolation by RNA Purification (ChIRP). While formaldehyde captures a broader range of interactions, it risks crosslinking proximal proteins nonspecifically, whereas UV crosslinking provides higher specificity but lower efficiency. Recent advancements like ​CARPID (CRISPR‐assisted RNA–protein interaction detection) bypass traditional crosslinking by leveraging RNA–targeting CRISPR/dCas13 to guide proximity‐labeling enzymes directly to endogenous RNAs, preserving native interactions. These methods collectively address challenges such as RNA folding complexity, low‐abundance RNA detection, and artifact minimization, making RNA‐centric approaches indispensable for unraveling RNA–protein networks in health and disease.

#### 2.1.1 Hybridization‐Based RNA Pulldown

In 2006, a method called PNA‐assisted identification of RBPs (PAIR) was developed by Dr. James Eberwine's laboratory to identify RBPs that interact with RNA of interest in living cells.^[^ ^26^, ^27^ ^]^ This approach utilizes peptide nucleic acids (PNAs) coupled with a cell‐penetrating peptide (transportan 10, TP10) to target specific mRNA regions.^[^ ^28^, ^29^ ^]^ The PNAs also contain a photoactivatable compound (Bpa) that crosslinks nearby proteins upon exposure to UV irradiation. After then, RNA–protein complexes are isolated using biotinylated sense oligonucleotide coupled to streptavidin beads for subsequent mass spectrometric (MS) analysis. The development of PAIR provides a valuable framework for mapping RBPs interacting with RNA molecules in vivo. Subsequently, ChIRP has been developed by leveraging the hybridization‐based RNA pulldown of crosslinking agents, which stabilized the RNA–chromatin complex to map the chromatin/protein interactomes of RNA within their native context in 2011.^[^ ^8^, ^30^, ^31^ ^]^ Similar to the ChIRP‐MS method, other hybridization‐based methods like capture hybridization analysis of RNA targets (CHART),^[^ ^32^, ^33^ ^]^ identification of direct RNA interacting proteins (iDRiP),^[^ ^10^, ^34^ ^]^ and RAP‐MS^[^ ^9^ ^]^ were also developed to study the RNA–protein interactions.

Despite the conceptual similarity among these methods, they differ in several key steps such as cell crosslinking, types of probes used, hybridization conditions, and elution methods (**Table** **1**). For example, the ChIRP and CHART methods typically use formaldehyde and glutaraldehyde to fix cells, while iDRiP and RAP‐MS utilize UV irradiation at 254 nm, a short‐range crosslinker, which is more likely to highlight direct RNA–protein interactions. Notably, RAP‐MS sets itself apart by using longer biotinylated antisense DNA or RNA probes of 90–120 nucleotides, compared to the typical 20–25 nucleotides. This leads to more stable hybrids, allowing RNA complex purification even under harsh conditions and enhancing the signal‐to‐noise ratio. Among these four hybridization‐based methods, ChIRP is the most widely utilized approach for systematically identifying RNA‐associated proteins. Leveraging short, biotinylated tiling oligonucleotides that hybridize to a target RNA, ChIRP‐MS enables the efficient isolation of endogenous RNA–protein complexes from crosslinked cellular lysates. The associated proteins are subsequently identified through MS, providing high‐resolution insights into the composition of RNA interactomes. Its compatibility with formaldehyde crosslinking and the straightforward design of probes make ChIRP‐MS particularly attractive for broad applications across diverse biological systems.^[^ ^35^ ^]^ To date, this technique has been successfully employed in studying a wide range of RNA species—including long noncoding RNAs (lncRNAs),^[^ ^36^, ^37^, ^38^ ^]^ viral RNAs,^[^ ^39^, ^40^, ^41^ ^]^ and chromatin‐enriched transcripts.^[^ ^42^, ^43^, ^44^ ^]^ CHART improves the specificity of RNA–protein interaction mapping by utilizing RNase H mapping to precisely identify accessible regions on the target RNA for probe design, thereby improving signal‐to‐noise ratios. However, this requirement for prior structural knowledge of the RNA limits its applicability and accessibility, which may contribute to its relatively lower adoption compared to more user‐friendly methods such as ChIRP.^[^ ^32^, ^45^ ^]^ iDRiP combines UV crosslinking with pools of short antisense DNA probes to selectively enrich RNAs along with their directly interacting proteins, enabling the capture of dynamic and transient RNA–protein complexes. Despite its relatively complex protocol,^[^ ^46^ ^]^ iDRiP has proven valuable in several studies, such as lncRNAs XIST^[^ ^10^ ^]^ and TERRA.^[^ ^46^, ^47^ ^]^ RAP‐MS distinguishes itself through the use of long, biotinylated antisense probes coupled with stringent hybridization conditions, providing high specificity and sensitivity in detecting RNA‐binding proteins.^[^ ^9^ ^]^ Although it demands substantial input material and involves a technically complex workflow, RAP‐MS has been successfully applied to identify protein interactors of several lncRNAs, including XIST,^[^ ^9^ ^]^ ALOX5,^[^ ^48^ ^]^ and DUBR,^[^ ^49^ ^]^ thereby offering valuable insights into their regulatory roles in diverse biological processes. Together, these methods provide complementary approaches, with method selection best guided by factors such as RNA abundance, localization, and the desired resolution of interaction mapping.

**Table 1:** Comparison of hybridization‐based RNA pulldown approaches.

Method	Crosslinkers	Probes	Advantages	Limitations	Refs.
PAIR	Ultraviolet 254 nm	Biotinylated sense DNA and PNA	Dynamic RBP mapping in living cells	Requires PNA design and optimization	[26, 27]
ChIRP	Formaldehyde, glutaraldehyde	Biotinylated antisense DNA (20–25 nt)	Easy to handle, user‐friendly	Limited in nuclear compartment	[8, 30]
CHART	Formaldehyde, glutaraldehyde	Biotinylated antisense DNA (20–25 nt)	High specificity	Needs RNase‐H mapping assay for probe design	[32, 33]
iDRiP	Ultraviolet 254 nm	Biotinylated antisense DNA (20–25 nt)	Identifying direct interactions	Difficult for RNAs in low abundance	[10, 34]
RAP‐MS	Ultraviolet 254 nm	Biotinylated antisense DNA/RNA (90–120 nt)	High specificity and signal‐to‐noise ratio	Difficult for RNAs in low abundance	[9]

The utilization of these hybridization‐based approaches provides various advantages in the comprehensive profiling and investigation of RNA–protein interactions. The most significant advantage is its high specificity, which allows for the accurate targeting of the functional elements of RNAs domain by domain.^[^ ^50^, ^51^ ^]^ Recently, to decrease the expense on synthesizing multiple biotinylated oligos that span the full length of RNA, a novel approach termed multiple oligo assisted RNA pulldown via hybridization (MORPH) has been introduced.^[^ ^52^ ^]^ MORPH uses a single biotinylated oligonucleotide to capture all antisense oligonucleotides, which effectively bind to the target RNA associated with chromatin. Consequently, this application of the MORPH method enhances the identification of RBPs in a manner that is not only more cost‐effective, but also more efficient.

#### 2.1.2 Proximity Labeling‐Based Protein Tagging

While hybridization‐based methods can capture both direct and indirect interactions, offering a comprehensive perspective on RNA–protein complexes, they come with inherent limitations, including dependence on UV or chemical crosslinking, which can introduce biases and noise. Consequently, researchers have opted for an alternative approach to identify RBPs by utilizing a proximity protein tagging system known as Proximity‐dependent biotin identification (BioID), designed to label proteins located near a specific RNA molecule within a living cell environment.^[^ ^53^ ^]^ Biotin is generally used in the labeling process, serving as a handle for subsequent isolation of tagged proteins with streptavidin beads.^[^ ^54^ ^]^ Different proximity labeling strategies leveraging either engineered biotin ligases activated by high concentration of biotin or enzymes like engineered ascorbate peroxidase APEX2 to transform an inert small‐molecule substrate into a short‐lived highly reactive species^[^ ^55^, ^56^, ^57^, ^58^ ^]^ (**Table** **2**). For example, APEX2 catalyzes oxidation of biotin‐tyramide by hydrogen peroxide (H_2_O_2_) and generates short‐lived radicals around the peroxidase. Efficient protein tagging is thus restricted in close proximity to APEX2 that has been brought to the RNA of interest with different strategies.

**Table 2:** Overview of proximity labeling enzymes.

Enzyme	Type		Size [kDa]	Labeling time	Modification sites	Refs
BioID		Biotin ligase	35	18 h	Lys	[57]
BioID2		Biotin ligase	27	18 h	Lys	[59]
TurboID		Biotin ligase	35	10 min	Lys	[58]
miniTurboID		Biotin ligase	28	10 min	Lys	[58]
BASU		Biotin ligase	35	1 min	Lys	[60]
AirID		Biotin ligase	35	1 h	Lys	[61]
APEX		Peroxidase	28	1 min	Tyr, Trp, His, Cys	[62]
APEX2		Peroxidase	28	1 min	Tyr, Trp, His, Cys, guanosine	[55, 63]
pafA		Pup ligase	60	24–36 h	Lys	[64]

Several methods that employ the proximity labeling technique are available, each varying in how the tagging enzyme is positioned near the target RNA (**Table** **3**).

**Table 3:** Comparison of proximity labeling‐based protein tagging approaches.

Method	Crosslinkers		Probes	Advantages	Limitations	Refs.
RaPID	No	No		Captures transient, weak interactions	Requires heterologous RNA expression	[60]
RNA‐BioID	No	No		Be able to track endogenous RNA	Requires genetic engineering	[65]
Hypro‐MS	Dithiobis (succinimidyl propionate)	DIG‐labeled antisense DNA		No genetic engineering	Requires cell crosslinking	[66, 67]

***RaPID*** *(RNA–Protein Interaction Detection)*: In this method, both RNA of interest and biotin‐ligase are ectopically expressed in the living cells.^[^ ^60^ ^]^ The targeted RNA is flanked by two BoxB stem loops, and the biotin‐ligase BirA* or BASU is fused to a 22‐amino‐acid λN peptide which exhibits a notable and specific affinity for BoxB stem loops, allowing the recruitment of biotin ligase to tag protein adjacent to the RNA. Then, in the presence of high concentrations of biotin, biotin ligase is activated, enabling it to label proximal proteins within an approximate range of 10 nm. The RaPID method enables unbiased and comprehensive identification of proteins that interact with a specific RNA of interest under physiological conditions. In addition, it allows for the detection of transient or low‐affinity interactions, which may have been overlooked by traditional methods. These benefits offer a more accurate and holistic understanding of the role of RNA–protein interactions in both cellular functionality and disease pathogenesis.^[^ ^60^ ^]^ For example, the RaPID technique has been used to evaluate the impact of disease‐associated point mutations on RNA–protein interactions and to identify host proteins that associate with Zika virus RNA, thereby elucidating molecular mechanisms relevant to pathogenesis and viral replication.^[^ ^60^ ^]^ However, one limitation of RaPID is the requirement for exogenous expression of BoxB‐tagged RNA, which may potentially perturb the native localization or function of the RNA, thereby introducing artifacts. Nevertheless, the method remains a powerful tool for dissecting dynamic RNA–protein interactions with high spatial and temporal resolution.

*RNA‐BioID*: To study the endogenous RNA, RNA‐BioID method involves integration of the BoxB sequence or alternatively MS2 hairpin RNA aptamers to the genomic locus of the RNA gene of interest with genome editing tools such as CRISPR/Cas9.^[^ ^65^ ^]^ The biotin ligase BirA* is fused to MS2 coat protein (MCP), which can specifically bind to the MS2‐containing RNA. Upon activation by high concentration of biotin, RNA‐adjacent proteins become biotinylated which eases subsequent purification and MS‐identification. Similar to RaPID, RNA‐BioID can be performed in living cells without the need for crosslinking. The number of potential interactors identified using RNA‐BioID is significantly higher than the number of proteins identified by hybridization‐based approaches, which may be attributed to the greater sensitivity of proximity labeling and its ability to capture transient interactions that might be missed by traditional methods that require crosslinking. For instance, RNA‐BioID has been effectively applied to investigate the dynamic interactome of endogenous β‐actin mRNA in mouse embryonic fibroblasts. By inserting MS2 binding sites into the 3′ UTR of β‐actin and tethering a BirA* via an MCP, the study enabled proximity‐dependent labeling of proteins associated with the mRNA under native conditions. This approach uncovered more than 60 previously unidentified RBPs associated with β‐actin, many of which exhibited condition‐specific binding dynamics under serum‐induced cell migration.^[^ ^65^ ^]^

Despite their versatility, both proximity labeling approaches rely on the ectopic integration of special RNA aptamers such as BoxB stem loops or MS2 into the target RNA, potentially affecting its native structure or binding proteins. Moreover, either the ectopic expression or genome editing requires transfection of plasmid into living cells, posing considerable challenges for their application to difficult‐to‐transfect cell types, nonmodel organisms, or clinical samples. It is of paramount importance to optimize both the design and expression levels of the fusion proteins used in these approaches. The optimization is necessary to not only diminish the labeling background, but also to curtail the likelihood of cytotoxicity and the emergence of mislocalization artifacts.

*HyPro‐MS*: To tackle these challenges, the HyPro‐MS method has been developed, merging the advantages of hybridization‐based RNA targeting with proximity labeling‐based protein tagging to enable the identification of RBPs in genetically unmodified samples.^[^ ^66^ ^]^ HyPro‐MS method involves hybridizing digoxigenin‐labeled antisense probes to target RNA in fixed and permeabilized cells. Then, a purified recombinant HyPro enzyme, which is a fusion of APEX2 and a digoxigenin‐binding domain, is recruited to these digoxigenin‐labeled probes. Upon the addition of biotin‐phenol and H_2_O_2_, the HyPro enzyme biotinylates nearby proteins, facilitating the identification of RBPs in their native cellular context without genetic modifications. In HyPro‐MS method, the use of digoxigenin‐labeled probes ensures specific binding to the target RNA, and the compact design of the HyPro enzyme allows for efficient proximity labeling with minimal background. Unlike previous proximity labeling‐based approaches that require the expression of recombinant enzymes in living cells, HyPro‐MS does not require any genetic engineering. Instead, it can be efficiently delivered to the RNA of interest in cells and achieve the labeling goal by utilizing the pairing characteristic of a digoxigenin‐labeled probe and the proximity labeling activity of purified HyPro enzyme. Notably, the use of a pre‐designed HyPro enzyme can reduce the time and effort needed to optimize the conditions for different experimental setups, benefiting the broad application of the HyPro method in various biological contexts and for different types of RNA, including those expressed at low levels. For instance, HyPro‐MS has been successfully applied to the ncRNAs 45S, NEAT1, and PNCTR, which are expressed at markedly different levels, thereby demonstrating its broad applicability across diverse RNA classes.^[^ ^66^ ^]^ These applications underscore the potential of HyPro‐MS in uncovering functional RNA–protein interaction landscapes without the need for genetic manipulation, which is particularly advantageous for working with clinical samples or primary cells. To ensure the reliability and accuracy of HyPro‐MS, meticulous RNA probe design is critical. One of the primary challenges lies in the complex secondary structures of target RNAs, which can significantly impede hybridization efficiency. Moreover, the choice and optimization of crosslinking methods are crucial, as suboptimal crosslinkers may fail to capture transient or indirect interactions and may introduce nonspecific protein associations. Overcoming these limitations requires strategic probe design that accounts for RNA structural features, along with carefully optimized crosslinking protocols to enhance the specificity and sensitivity of protein–RNA interaction detection. Overall, HyPro‐MS definitely expanded the scope of RNA research by enabling the analysis of RNA interactomes with high specificity and sensitivity in genetically unperturbed samples, facilitating a deeper understanding of RNA functions and interactions within the cells.

#### 2.1.3 CRISPR‐Assisted Proximity Labeling

The discovery of CRISPR/Cas9 system has significantly expanded the possibilities for genome studies and has driven rapid advancements in biomedicine.^[^ ^68^ ^]^ In a similar breakthrough, the RNA‐targeting CRISPR/Cas13a system, identified in 2016, quickly emerged as a pivotal development in the CRISPR field.^[^ ^69^ ^]^ This discovery was soon followed by the identification of additional CRISPR/Cas13 variants, including CRISPR/Cas13b/c,^[^ ^70^ ^]^ CRISPR/Cas13d,^[^ ^71^, ^72^ ^]^ CRISPR/Cas13e/f/g/h/i,^[^ ^73^ ^]^ and CRISPR/Cas13X/Y.^[^ ^74^, ^75^ ^]^ Like DNA‐targeting CRISPR/Cas systems, CRISPR/Cas13 is guided by a CRISPR RNA (crRNA) that directs the system to cleave RNA targets containing complementary protospacers. However, unlike many DNA‐targeting CRISPR/Cas systems that require a protospacer adjacent motif (PAM), Cas13 does not exhibit a significant preference for protospacer flanking sequences (PFS) in eukaryotic cells. This lack of PFS preference allows Cas13 proteins to target a broader range of RNA sequences. The Cas13 proteins typically contain two higher eukaryote and prokaryote nucleotide‐binding (HEPN) RNase domains, which together form a single catalytic site responsible for cleaving target RNAs.^[^ ^76^, ^77^, ^78^ ^]^ In addition, the Cas13 proteins have RNase activity that processes their own crRNAs from a pre‐crRNA array composed of multiple spacers and direct repeats (DRs), allowing for multiplexed targeting by enabling the expression of multiple crRNAs from a single transcript.^[^ ^71^, ^79^ ^]^

Currently, the development of RNA‐targeting CRISPR/Cas13 system has revolutionized the precise manipulation of endogenous RNAs, leading to significant advancements in our understanding of RNA regulation and the development of RNA‐based diagnostic and therapeutic technologies.^[^ ^80^ ^]^ Specifically, the catalytically inactive form of the Cas13 enzyme, usually known as dead Cas13 (dCas13), has been widely used for various RNA targeting and manipulation applications. Despite its catalytic inactivity, dCas13 retains its pre‐crRNA processing RNase activity, which enables it to maintain its multiplexing capability. When assembled with a guide RNA (gRNA), dCas13 can be precisely directed to RNA sequences complementary to the gRNA, enabling it to function as a programmable RBP that navigates fused effector protein in close proximity to the target RNA. Taking the advantage, various methods utilizing different versions of the CRISPR/Cas13 system and various proximity labeling enzymes to identify the RBPs of a range of lncRNAs have come up almost at the same time, including CRISPR‐assisted RNA–protein interaction detection (CARPID),^[^ ^11^ ^]^ CRISPR‐based RNA‐United Interacting System (CRUIS),^[^ ^81^ ^]^ dCas13d‐dsRBD‐APEX2,^[^ ^82^ ^]^ CRISPR‐based RNA proximity proteomics (CBRPP),^[^ ^83^ ^]^ and RNA proximity labeling (RPL) Cross‐linking and immunoprecipitation^[^ ^84^ ^]^ (**Table** **4**), demonstrating the pressing need and great feasibility of such approaches to identify the binding proteins of specific endogenous RNAs of interest in living cells.

**Table 4:** Comparison of CRISPR‐assisted proximity labeling approaches.

Method	RNA navigator	Proximity labeling enzyme	Size [kDa]	Examples of tested RNA	Refs.
CRUIS	dCas13a	PafA	≈200	NORAD	[81]	
CARPID	dCas13d	BASU	≈147	XIST, MALAT1, DANCR	[11]	
dCas13d‐dsRBD‐APEX2	dCas13d	APEX2	≈150	hTR	[82]	
CBRPP	dCas13b	BioID2	≈155	ACTB, NORAD	[83]	
RPL	dCas13b	APEX2	≈156	U1, Poly(A) tail	[84]	

*CARPID*: Similar to a strategy that utilized CRISPR/dCas9 to direct proximity labeling enzymes to specific genomic loci for identification of DNA‐binding proteins,^[^ ^85^, ^86^ ^]^ the CARPID method has been developed to leverage CRISPR/dCas13 for RNA targeting and enables fused BASU biotin ligase for proximity labeling to identify binding proteins of specific endogenous RNAs.^[^ ^11^ ^]^ Through integrating CRISPR‐based RNA targeting with proximity labeling, the CARPID method overcomes the requirement of crosslinking and enables the identification of RBPs in living cells. By applying the CARPID method, the binding proteins of three endogenous lncRNAs with different expression levels and subcellular localizations, including XIST, MALAT1, and DANCR, were successfully identified.^[^ ^11^ ^]^ Furthermore, CARPID has been effectively employed to elucidate the functional role of the lncRNA TUG1 in R‐loop resolution during replication stress in cancer cells.^[^ ^87^ ^]^ In this study, CARPID enabled the identification of proteins in close proximity to TUG1, including DHX9 and RPA32, both of which were found to localize within ≈10 nm of the RNA in the nucleus, thereby contributing to the maintenance of genome stability and supporting cancer cell proliferation. In addition, to investigate the role of lncRNAs in TGF‐β‐induced epithelial‐mesenchymal transition (EMT) in breast cancer, CARPID was utilized in MDA‐MB‐231 cells to identify the protein interactors of LITATS1 and LETS1. The binding partners of LITATS1, TβRI, and SMURF2,^[^ ^88^ ^]^ as well as NFAT5, identified as a key interactor of LETS1,^[^ ^89^ ^]^ were found to play critical roles in the regulation of cancer cell migration. Moreover, to elucidate the molecular mechanism by which the circular RNA ciTRAN modulates HIV‐1 transcription, CARPID was employed in HIV‐1‐infected T cells to identify ciTRAN‐associated proteins. This analysis revealed that SRSF1, a splicing factor known to repress HIV‐1 transcription, interacts with ciTRAN, suggesting that HIV‐1 may hijack ciTRAN to sequester SRSF1 and promote viral gene expression.^[^ ^90^ ^]^ One of the most critical steps in CARPID analysis is to control the specificity of the targeted binding of dCas13 and gRNA. The binding specificity of dCas13 is intricately linked to the design of gRNAs. Poorly designed gRNAs may result in off‐target interactions, particularly in regions of RNA with high structural complexity. Optimization strategies include the selection of target regions with minimal secondary structure, the use of multiple gRNAs to enhance coverage, and the employment of high‐fidelity Cas13 variants to mitigate off‐target effects.

Besides the targeting specificity, the delivery and expression efficiency of the dCas13‐BASU is another critical issue. All of these CRISPR‐assisted proximity labeling approaches require the expression of recombinant enzymes in living cells, which constrains their broad applicability to various cell types and tissues that are difficult to transfect. In addition, unassembled enzymes that failed to bind to the target RNAs can produce nonspecific signals, thereby introducing background noise. To further circumvent these limitations, one may consider changing the plasmid‐mediated expression of CARPID components to the RNP delivery systems^[^ ^91^ ^]^ in which recombinant CARPID protein can be generated in *E. coli* and assembled with synthesized sgRNA in vitro. This advancement presents expanded opportunities for the exploration of RNA–protein interactions in a wider array of biological contexts and intricate cellular environments. The efficacy of delivering RNP complexes varies depending on the cell type. While lipofection or electroporation can be effective in specific cell lines, primary cells or in vivo systems may necessitate the use of viral delivery systems or nanoparticle formulations. Transient delivery of RNPs is typically preferred to minimize long‐term off‐target effects, although the efficiency of cellular uptake remains a concern that often requires empirical optimization.

Different RNA‐centric approaches offer distinct advantages depending on experimental goals. UV crosslinking‐based methods like iDRiP excel at capturing direct RNA–protein interactions through zero‐length covalent bonds, as demonstrated by its high specificity for telomeric TERRA and spliceosomal U1 snRNP complexes, while minimizing mitochondrial contaminants seen in formaldehyde‐based ChIRP. In contrast, chemical crosslinking methods (ChIRP, CHART) preserve broader macromolecular networks, enabling identification of both direct and indirect interactors through formaldehyde‐mediated DNA–RNA–protein bridges, albeit with increased background noise. Enzymatic proximity labeling techniques (CARPID, RaPID) leverage CRISPR‐guided or RNA‐tagging systems to biotinylate neighboring proteins, offering advantages for low‐abundance RNAs but potentially capturing indirect associations. Ultimately, method selection should consider RNA abundance, interaction dynamics, and desired resolution of direct versus proximal associations.

While many sequencing‐based methods could be advanced to study the RBP‐RNA interaction in a single cell resolution, it is still very challenging to develop a RNA‐centric method, which mostly depends on MS technique, to identify binding proteins in a single cell. However, the development of MS‐based single‐cell proteomics shows great progress in addressing these challenges, enabling precise identification of specific biotinylation sites in proteins. This advancement has the potential to transform the detection of RNA‐binding proteins at single‐cell resolution with enhanced precision and sensitivity.

### 2.2 Protein‐Centric Methods

**Figure 2:**
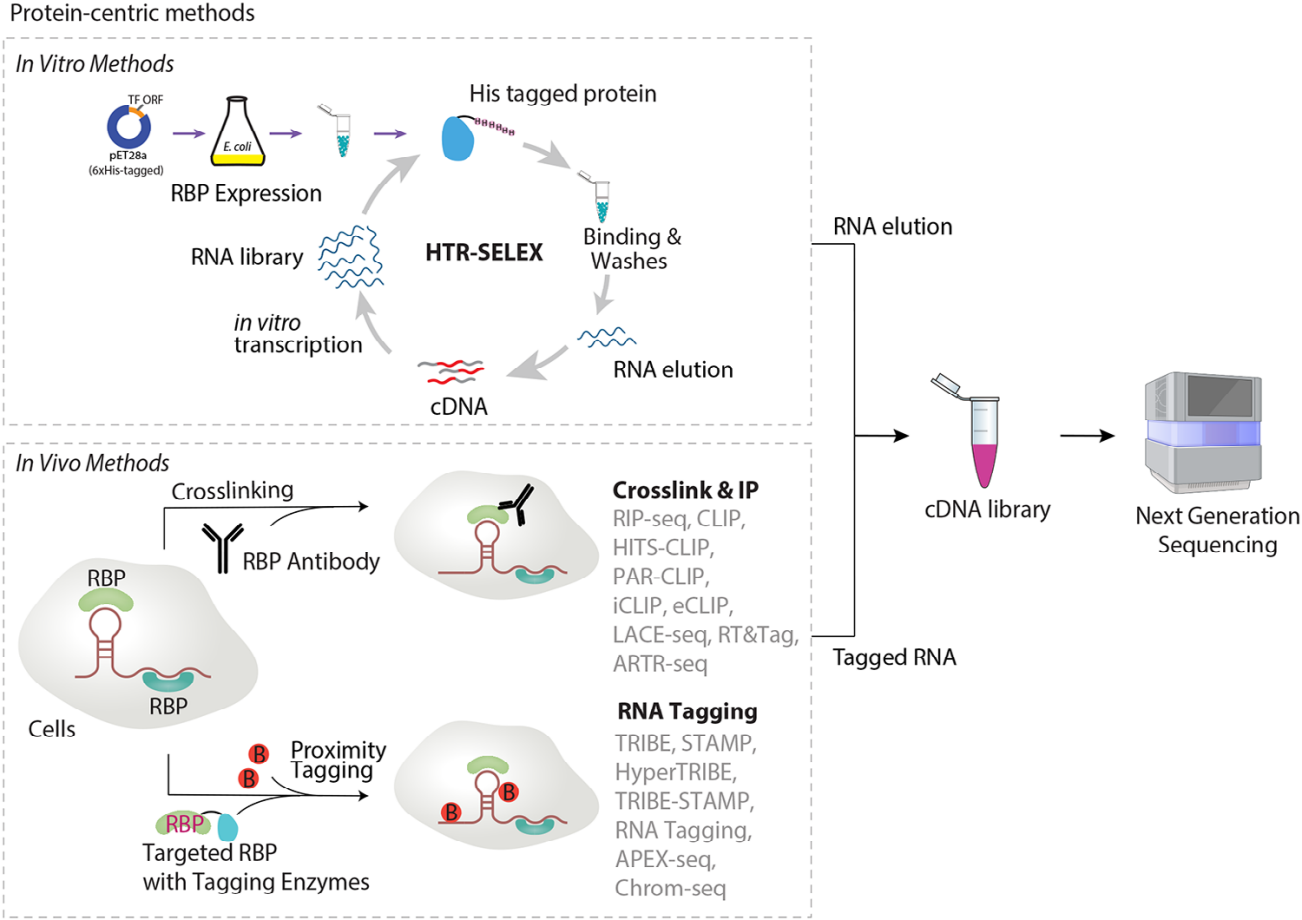
Schematic overview of protein‐centric methods. in vitro methods (upper): His‐tagged fusion proteins are purified and incubated with a barcoded RNA library. After washing, RNA ligands bound by the proteins are recovered by reverse transcription and PCR for subsequent enrichment cycles. in vivo methods (lower): Immunoprecipitation‐based RNA identification methods often employ crosslinkers to stabilize RNA‐protein interactions. The RNA bound to the target protein is then captured using specific antibody. In RNA tagging‐based approaches, target proteins are expressed as fusions with RNA‐modifying enzymes such as ADAR, APOBEC1, PUP, or APEX2, which mark nearby molecules. The labeled RNA are enriched for library preparation and analyzed by sequencing.

Transitioning from RNA‐centric approaches that map RNA interactomes by targeting RNA molecules, protein‐centric methods shift the focus to RBPs to systematically identify their associated RNA targets and binding sites. While RNA‐centric techniques excel in profiling RNAs and their interacting partners, protein‐centric strategies are indispensable for deciphering the roles of RBPs in post‐transcriptional regulation, such as splicing, stability, and translation. These methods broadly fall into three categories: (1) high‐throughput in vitro assays (e.g., HTR‐SELEX, SEQRS), which screen RBP binding specificities using synthetic RNA libraries; (2) immunoprecipitation‐based approaches (e.g., RIP, CLIP, LACE‐seq), which crosslink and purify RBP‐RNA complexes in vivo for sequencing; and (3) proximity‐tagging techniques (e.g., Chrom‐seq, TRIBE), which exploit editing enzymes or proximity ligases to label RNAs proximal to RBPs in living cells (**Figure** **2**).

Protein‐centric methods have evolved to address key challenges, such as capturing transient interactions and resolving binding sites at nucleotide resolution. For instance, Cross‐linking and immunoprecipitation followed by sequencing (CLIP‐seq) uses UV crosslinking to stabilize RBP–RNA interactions, followed by immunoprecipitation and sequencing to map binding sites with high precision. Meanwhile, proximity‐based innovations like Targets of RNA‐binding proteins identified by editing (TRIBE) fuse RBPs with RNA‐editing enzymes (e.g., ADAR) to introduce mutations in bound RNAs, enabling identification of dynamic interactions without crosslinking. These approaches complement RNA‐centric strategies by providing mechanistic insights into how RBPs regulate RNA fate, often with cell‐type or subcellular specificity.

#### 2.2.1 in vitro High‐Throughput Analysis

RNA‐SELEX (systematic evolution of ligands by exponential enrichment) is a classic in vitro method for identifying RNA sequences that bind to a target protein with high affinity. This technique involves iterative rounds of selection and amplification to evolve RNA molecules that exhibit strong binding affinity to the target protein.^[^ ^92^, ^93^ ^]^ With the advancement of Next‐Generation Sequencing (NGS), high‐throughput sequencing of RNA substrate selectivity landscapes (SEQRS), and high‐throughput RNA‐SELEX (HTR‐SELEX) methods have been developed based on the principles of the classic RNA‐SELEX.^[^ ^94^, ^95^, ^96^, ^97^ ^]^ These two approaches leverage the power of NGS to enable high‐throughput analysis of the RNA binding specificities of RBPs. In comparing SEQRS and HTR‐SELEX, it is noted that SEQRS utilizes an RNA pool with 20 randomized nucleotides, whereas HTR‐SELEX employs an RNA pool with 40 randomized nucleotides. This difference in the length of randomized nucleotides in the RNA pool impacts the diversity and complexity of the RNA library available for selection in each round (**Table** **5**). By applying HTR‐SELEX, researchers have identified the binding specificities of 86 human RBPs, revealing that many RBPs exhibit a preference for binding structured RNA motifs and can interact with multiple distinct sequences.^[^ ^95^ ^]^ During the COVID‐19 pandemic, HTR‐SELEX has been applied to characterize the RNA binding specificity of the nucleocapside (N) proteins of the coronaviruses, shedding light on the mechanisms of viral genome packaging. This knowledge holds substantial implications for advancing the development of potent therapeutics aimed at combating viral infections.^[^ ^98^ ^]^

**Table 5:** Comparison of Immunoprecipitation‐based RNA identification approaches.

Methods	Crosslinkers	Advantages	Limitations	Refs.
SEQRS	No	High‐throughput analysis in vitro	Lacks cellular environment	[94]
HTR‐SELEX	No	RNA pool with longer nucleotides	Lacks cellular environment	
RIP‐Seq	Formaldehyde, Ultraviolet 254 nm	User‐friendly, without genetic manipulation	Relatively high background, low resolution	[134]
CLIP	Ultraviolet 254 nm	Able to map exact RNA binding sites	Low efficiency of crosslinking	[100, 135]
HITS‐CLIP	Ultraviolet 254 nm	High‐throughput analysis in vivo	Requires millions of cells	[101]
PAR‐CLIP	Ultraviolet 365 nm plus photoactivatable nucleosides	Higher RNA recovery efficiency	Requires millions of cells	[102]
iCLIP	Ultraviolet 254 nm	Single nucleotide resolution	Requires millions of cells	[103]
eCLIP	Ultraviolet 254 nm	Enables large‐scale and robust profiling	Needs advanced bioinformatics	[104]
LACE‐seq	Ultraviolet 254 nm	Single nucleotide resolution with low‐input cells	Difficult to detect transient interactions	[117]
RT&Tag	No	No need crosslinking, Simplicity	Only available for polyA‐specific RNAs	[128]
ARTR‐seq	Formaldehyde	High resolution with low‐input cells	Depends on the quality of antibodies	[130]

#### 2.2.2 Immunoprecipitation‐Based RNA Identification

For in vivo protein‐centric approaches, co‐immunoprecipitation of RBP‐associated RNA serves as the pioneering technique that comprehensively maps the locations where proteins interact with RNA at a genome‐wide level.^[^ ^99^ ^]^ It does not require prior knowledge of the RNA sequences, enabling the discovery of novel RNA–protein interactions (Table 5).

*RIP‐seq (RNA Immunoprecipitation followed by Sequencing)*: The process of RIP‐seq involves antibody‐based immunoprecipitation of an RNA‐binding protein capturing its associated RNA from cell lysates for subsequent sequencing and identification, which is relatively straight‐forward and easy to handle. To stabilize RNA–protein (RNP) interactions during the immunoprecipitation process, crosslinking the RNP complex with UV or formaldehyde is an option that can significantly improve the efficiency of the capture. Optimizing crosslinking conditions is essential in this method to achieve efficient stabilization of RNA–proteins interactions without causing excessive damage to RNA or proteins. While the RIP‐seq method is effective for identifying potential target RNAs of a protein, it could not be easily conducted in a high‐throughput manner given the limited availability of high quality of antibodies. Another curb of RIP is the suboptimal resolution that is inadequate to pinpoint the exact binding sites on the RNAs.

*CLIP and its derived methods*: To overcome the resolution issue and map the precise binding sites of proteins on RNA molecules, a series of (CLIP)‐derived methods have been developed. In comparison to RIP method, it introduces one step of RNase‐treatment to digest unprotected RNA regions after crosslinking (e.g., RNase I in iCLIP), leaving only protein‐bound RNA fragments (≈20–60 nt). This enables ​single‐nucleotide resolution by pinpointing exact binding sites, which allows researchers to gain a more accurate understanding of RNA–protein interactions and their regulatory mechanisms.^[^ ^100^ ^]^ To date, several CLIP‐derived methods have been developed to further enhance both resolution and specificity by optimizing crosslinking and library preparation efficiency, such as HITS‐CLIP (standing for High‐Throughput Sequencing of RNA),^[^ ^101^ ^]^ PAR‐CLIP (standing for Photoactivatable‐Ribonucleoside‐Enhanced CLIP),^[^ ^102^ ^]^ iCLIP (standing for Individual‐nucleotide resolution CLIP),^[^ ^103^ ^]^ and eCLIP (standing for enhanced CLIP).^[^ ^104^ ^]^ These advancements have significantly improved the utility of CLIP methods and deepened our insights into RNA biology by offering more accurate and specific data on RNA–protein interactions, as comprehensively summarized in these reviews.^[^ ^105^, ^106^, ^107^ ^]^

These CLIP methods offer distinct methodological advantages tailored to different research needs. HITS‐CLIP provides robust, transcriptome‐wide interaction mapping, although it often suffers from moderate resolution due to imprecise crosslinking sites. It has proven instrumental in uncovering the regulatory roles of Argonaute proteins in miRNA‐mediated gene silencing,^[^ ^108^, ^109^ ^]^ as well as mapping NOVA protein binding sites involved in alternative splicing regulation in the nervous system.^[^ ^101^, ^110^ ^]^ PAR‐CLIP enhances both crosslinking efficiency and resolution by incorporating photoactivatable ribonucleoside analogs (e.g., 4‐thiouridine or 6‐thioguanosine) into nascent RNA, enabling detection of characteristic T‐to‐C transitions at binding sites. This makes it particularly suitable for studies requiring high‐confidence site identification, such as investigations of post‐transcriptional regulatory networks or RNA turnover.^[^ ^111^, ^112^ ^]^ iCLIP introduces a reverse transcription truncation signature, enabling precise mapping of protein–RNA interaction sites at single‐nucleotide resolution, making it ideal for dissecting fine‐scale regulatory mechanisms such as splicing regulation. This level of precision is critical for elucidating mechanisms of alternative splicing and transcript processing.^[^ ^113^, ^114^ ^]^ eCLIP, an optimized version of iCLIP developed under the ENCODE consortium, standardizes and enhances the iCLIP protocol by introducing size‐matched input controls and streamlined library preparation, increasing reproducibility and sensitivity while reducing input requirements. This makes eCLIP advantageous for high‐throughput applications across multiple RBPs, as exemplified by large‐scale profiling efforts in human cell lines.^[^ ^115^, ^116^ ^]^ Accordingly, the selection of a CLIP‐seq variant should be guided by the desired resolution, sample limitations, and biological context, with iCLIP favored for fine‐scale mechanistic insights, PAR‐CLIP for high‐confidence target site mapping, and eCLIP for systematic, large‐scale analyses.

*LACE‐seq (linear amplification of complementary DNA ends and sequencing)*: While CLIP‐derived methodologies are capable of accurately pinpointing the RNA binding sites of target proteins, they often require a substantial number of cells and involve stringent conditions that have the potential to disturb native interactions. To overcome these limitations and enable precise mapping of RNA–protein interactions with fewer cells, LACE‐seq has been developed by Dr. Yuanchao Xue and collaborators.^[^ ^117^ ^]^ LACE‐seq method leverages RNA binding protein‐mediated reverse transcription termination on immunopurified protein–RNA complex, followed by linear amplification of the terminating cDNA ends. In LACE‐seq method, cells are crosslinked with UV‐C to preserve native RBP‐RNA interactions, followed by cell lysis and immunoprecipitation of the complexes using specific antibodies. The bound RNA is then fragmented on beads with micrococcal nuclease, and the 3′ ends are dephosphorylated and ligated with a 5′ pre‐adenylated linker. Reverse transcription is carried out on beads with a biotinylated primer containing a T7 promoter sequence, resulting in cDNA that terminates at crosslinking sites. The cDNA is subsequently polyadenylated, enriched through PCR amplification and in vitro transcription, and converted into sequencing libraries for single‐end deep sequencing. This advanced sequencing library preparation strategy allows LACE‐seq to achieve unbiased, high‐resolution mapping of RNA–protein interactions at a single‐nucleotide resolution using fewer cells, or even at the single‐cell level, significantly enhancing the study of dynamic RNA–protein interactions in germline and early embryo development.

LACE‐seq has proven to be a powerful tool for delineating the binding profiles and regulatory functions of RBPs in low‐input cellular systems. In studies focused on mammalian oocytes and early embryonic development, LACE‐seq enabled high‐resolution mapping of RBP binding sites for key regulators such as AGO2, MILI, DDX4, PTBP1, EIF4E1B, NAT10, LSM14B, and ZAR1/2.^[^ ^117^, ^118^, ^119^, ^120^, ^121^ ^]^ These insights have significantly advanced our understanding of the complex post‐transcriptional regulatory networks that govern oocyte development and maturation. To elucidate the molecular mechanisms of RBPs‐mediated spermatogenesis, LACE‐seq has been used to profile the binding events of RBPs in enriched spermatogonia, including SRSF1,^[^ ^122^ ^]^ SRSF2,^[^ ^123^ ^]^ SRSF10,^[^ ^124^ ^]^ RBM46,^[^ ^125^ ^]^ and CWF19L2.^[^ ^126^ ^]^ Collectively, these findings highlight the unique capability of LACE‐seq to investigate dynamic RNA–protein interactions in rare or developmental restricted cell types, offering critical insights into germ cell biology, early embryogenesis, and reproductive pathologies.

*RT&Tag*: Although CLIP and LACE‐seq can provide base resolution binding of RBP and RNA in vivo, it heavily relies on the UV‐mediated crosslinking to stabilize the binding which simultaneously introduces nonspecific noise. Inspired by the success of cleavage under targets and tagmentation (CUT&Tag) method, which eliminates the need for cell crosslinking and bypasses the immunoprecipitation process by tethering Tn5 transposase based tagmentation in situ,^[^ ^127^ ^]^ reverse transcribe and tagment (RT&Tag) has been developed by Dr. Kami Ahmad's laboratory in collaboration with Dr. Steven Henikoff to identify the binding RNA of target protein within native cellular context.^[^ ^128^ ^]^ By combining reverse transcription and Tn5‐mediated in situ tagmentation, the RT&Tag method efficiently identifies ​polyadenylated RNAs associated with specific proteins recognized by antibodies. In RT&Tag method, isolated cell nuclei are incubated with a primary antibody that specifically binds to the target protein. Subsequently, biotinylated oligo(dT)‐adapter‐B is recruited via a streptavidin‐conjugated secondary antibody, while protein A‐Tn5‐adapter‐A is concurrently engaged through binding to the same secondary antibody. in situ reverse transcription and tagmentation are then performed concurrently by the addition of reverse transcriptase. The cDNA will be sequenced for identification and quantitation of the RNA molecules associated with the protein of interest. Utilizing this proximity labeling strategy, the RT&Tag method simplifies the workflow for studying RNA–protein interactions without tedious precipitation and washing steps, enabling analysis with fewer cells or potentially a single cell broadly expanding its applicability.

The utility of RT&Tag has been demonstrated in *Drosophila* cells by capturing the ncRNA roX2 within the dosage compensation complex, as well as nascent transcripts associated with repressive histone modifications, illustrating its effectiveness in profiling functionally diverse RNA populations. In addition, RT&Tag has been employed to detect N6‐methyladenosine (m^6^A)‐modified mRNAs, highlighting its potential in linking epitranscriptomic regulation with transcriptional dynamics.^[^ ^128^ ^]^ More recently, RT&Tag was upgraded to profile RNA dynamics within nuclear compartments of human cell lines through combining RNA metabolic labeling, termed SLAM‐RT&Tag.^[^ ^129^ ^]^

*ARTR‐seq (Assay of RT‐based RBP binding site sequencing)*: Leveraging the combination of oligo(dT) primer‐initiated reverse transcription and Tn5 tagmentation of the resulting RNA/DNA heteroduplexes in isolated nuclei, RT&Tag proves to be particularly effective for the study of polyadenylated RNAs. However, its application is limited in the context of non‐polyadenylated RNAs and cytoplasmic RNA analysis.^[^ ^128^ ^]^ To overcome this limitation, ARTR‐seq method replaces oligo(dT)‐adapter with primers of random sequences in the in situ reverse transcription to generate cDNAs, which are labeled with biotinylated nucleotides, thus providing a more comprehensive analysis of RNA‐protein interactions.^[^ ^130^ ^]^ As a result, ARTR‐seq method enables the unbiased detection of RNA binding by proteins in both cytoplasm and nucleus, requiring as few as 20 cells or even a single tissue section. Moreover, an imaging step can be seamlessly integrated into the ARTR‐seq procedure, offering direct spatial information of RNA–protein interactions.^[^ ^130^ ^]^

In terms of applications, ARTR‐seq has been successfully employed to profile a variety of RBPs, including splicing factors such as PTBP1 and RBFOX2, as well as m⁶A readers like YTHDF1, YTHDF2, and YTHDC1. This method revealed compartment‐specific binding patterns, exemplified by the predominantly nuclear localization of YTHDC1 and the cytoplasmic association of YTHDF1/2.^[^ ^130^ ^]^ Furthermore, ARTR‐seq facilitated spatially resolved mapping of human transcripts within nuclear speckles, uncovering distinct localization patterns and their functional associations with splicing regulation.^[^ ^131^ ^]^ The ARTR‐seq data analysis revealed that transcripts stably enriched in nuclear speckles frequently contain retained introns and exhibit heightened sensitivity to speckle disruption, underscoring the role of nuclear speckles in both co‐ and post‐transcriptional regulation.^[^ ^131^ ^]^ These findings collectively highlight ARTR‐seq as a powerful tool for high‐resolution, low‐input, and spatially informative mapping of RNA‐protein interactions across both nuclear and cytoplasmic compartments.

In these immunoprecipitation‐based methods, the success of the experiments heavily relies on the selection and optimization of high‐quality antibodies. These antibodies must specifically and efficiently bind to the protein of interest without cross‐reacting with other proteins. However, the availability of high‐quality antibody has significantly limited access, impeding the wide applications of these approaches. Challenges include the commercially unavailability or discontinuation of certain antibodies and the potential for some antibodies to bind nonspecifically to other proteins, resulting in elevated background noise and compromised data integrity. To address this limitation, an alternative strategy is to genetically tag the protein of interest with an epitope tag (such as FLAG, HA, or Myc) using advanced CRISPR/Cas9 genome editing,^[^ ^132^, ^133^ ^]^ which however is both time‐ and labor‐demanding.

#### 2.2.3 RNA Tagging‐Based Methods

While co‐immunoprecipitation‐based methods have revolutionized RNA‐protein interaction mapping through crosslinking and antibody‐mediated capture, emerging proximity‐tagging strategies now offer complementary advantages by circumventing limitations such as antibody dependency, crosslinking artifacts, and low resolution in transient interaction detection. Proximity‐based methods leverage enzymatic tagging systems to label RNA–protein interactions in situ, enabling spatial precision and scalability. For instance, ​TRIBE employs RNA‐editing enzymes (e.g., ADAR fused to RNA‐binding proteins) to mark interacting RNAs with sequence‐specific mutations detectable via sequencing. Similarly, ​Chrom‐seq integrates engineered chromatin‐binding modules with APEX2 peroxidase to biotinylate RNAs proximal to specific epigenetic marks without crosslinking. These approaches bypass labor‐intensive immunoprecipitation steps while achieving single‐cell compatibility in some cases. Recent innovations like Particle‐templated instant partition sequencing (​PIP‐seq) further enhance throughput by combining proximity‐dependent biotinylation with droplet‐based workflows for transcriptome‐wide profiling.^[^ ^136^ ^]^ Collectively, proximity‐tagging methods expand the toolkit for studying dynamic RNA–protein networks, particularly in contexts where antibody availability or cell input constraints hinder traditional approaches (**Table** **6**).

**Table 6:** Comparison of RNA tagging‐based sequencing approaches.

Methods	Enzymes		Advantages	Limitations	Refs.
TRIBE	ADAR	Crosslinking and antibody‐free		Low editing efficiency	[137]
HyperTRIBE	ADAR2	Enhanced editing activity		Limited to double‐stranded RNA	[138]
STAMP	APOBEC1	Enable single‐stranded RNA editing		Limited to structured RNA	[146]
TRIBE‐STAMP	ADAR, APOBEC1	Simultaneously identify target RNAs		Technically difficult to handle	[149]
RNA Tagging	PUP	Provides quantitative binding information		Needs advanced bioinformatics	[152]
APEX‐seq/Chrom‐seq	APEX2	Controllable RNA tagging process		Limited to structured RNA	[63, 156, 157]

*TRIBE*: The TRIBE method has been developed by leveraging the RNA‐editing capability of the ADAR enzyme.^[^ ^137^ ^]^ The protein of interest is fused to the catalytic domain of an RNA editing enzyme ADAR. When the fusion protein binds to RNA, the ADAR component induces adenosine‐to‐inosine (A‐to‐I) editing at the site of interaction. These A‐to‐I editing events serve as markers, allowing target RNAs to be identified via deep sequencing. Compared to traditional immunoprecipitation‐based methods, TRIBE significantly simplifies the workflow and is particularly well‐suited to identify target RNAs within small numbers of living cells. Furthermore, the enhanced HyperTRIBE method, which uses a hyperactive variant of ADAR, exhibits substantially increased RNA editing activity and reduced local sequence preferences.^[^ ^138^ ^]^ This enhancement significantly boosts the sensitivity and efficiency of detecting RNA–protein interactions for HyperTRIBE, enabling transcriptome‐wide mapping of these interactions and holding the potential to achieve single‐cell resolution.^[^ ^139^ ^]^

TRIBE and HyperTRIBE have been successfully applied in multiple biological systems. For example, in Drosophila neurons, TRIBE was first used to identify in vivo RNA targets of the neuronal RBP Hrp48, revealing a broad spectrum of targets involved in neuronal function.^[^ ^137^ ^]^ Subsequently, HyperTRIBE was applied to map targets of FMRP (Fragile X Mental Retardation Protein) in the nervous system, uncovering key regulators of synaptic plasticity and neurodevelopment.^[^ ^138^ ^]^ These methods have also been adapted to mammalian systems, such as embryonic stem cells,^[^ ^140^, ^141^ ^]^ as well as to plants and microbial organisms,^[^ ^142^, ^143^, ^144^ ^]^ to define RNA targets of splicing regulators and translational repressors across diverse developmental and environmental contexts. Despite these advantages, TRIBE‐based methods have certain limitations. Deep sequencing is required to achieve sufficient coverage of editing events, increasing experimental cost. In addition, N‐ or C‐terminal fusions with ADAR may impair the native function or localization of certain RBPs. Moreover, ectopic overexpression of the fusion protein must be carefully optimized to prevent nonspecific editing and potential cellular toxicity.

*STAMP (surveying targets by APOBEC‐mediated profiling)*: ADAR‐mediated RNA editing is constrained by the limited availability of double‐stranded RNA regions proximal to protein binding sites.^[^ ^145^ ^]^ To overcome this limitation, the STAMP method has been developed.^[^ ^146^ ^]^ This approach substitutes ADAR with APOBEC1, a cytosine deaminase that facilitates RNA cytosine‐to‐uracil (C‐to‐U) conversion on single‐stranded RNA substrates.^[^ ^147^ ^]^ The increased likelihood of encountering APOBEC1 cytosine substrates enhances the sensitivity of the STAMP method, facilitating its integration with single‐cell capture techniques and providing high‐resolution insights into the dynamics of RNA–protein interactions within a single cell. A representative application of STAMP involved defining the transcriptome‐wide RNA‐binding landscape for a range of RBPs and ribosome subunits at the single‐cell resolution, such as RBFOX2, TIA1, RPS2, and RPS3.^[^ ^146^ ^]^ In addition, STAMP was used to reveal the transcriptome‐wide binding profiles of PABPN and PABPC, the nuclear and cytoplasmic poly(A)‐binding proteins. These findings demonstrate that transcripts exhibit differential associations with nuclear and cytoplasmic PABPs depending on their splicing status, poly(A)‐tail length, stability, and translation efficiency.^[^ ^148^ ^]^ Interestingly, the integration of STAMP with the TRIBE approach, termed TRIBE‐STAMP, has further enabled the simultaneous detection of individual RNA molecules bound by two different proteins. By fusing APOBEC1 and ADAR to distinct RBPs, this method revealed that individual mRNAs are often sequentially or concurrently targeted by multiple YTHDF proteins during their lifespan.^[^ ^149^ ^]^ This dual‐tagging strategy opens new avenues for investigating cooperative and competitive interactions between RBPs at the single‐molecule level, making TRIBE‐STAMP a powerful tool for dissecting the combinatorial logic of RNA regulation.^[^ ^150^ ^]^

*PUP‐Based RNA Tagging*: The poly(U) polymerase (PUP) is an enzyme that covalently adds uridines to the 3′ end of RNA molecules.^[^ ^151^ ^]^ Leveraging this capability, the RNA Tagging method has been developed to identify protein–RNA interactions in living cells.^[^ ^152^ ^]^ This method involves the expression of a fusion protein that combines a protein of interest with the PUP. Once expressed in cells, the fusion protein binds to target RNAs, and covalently adds uridines to their 3′ ends, creating U‐tags. Subsequently, the ectopically uridylated RNAs can be selectively reverse‐transcribed into cDNA for library preparation and sequencing to determine the RNA identities. Since the RNA tagging method relies on the covalent addition of uridines to the 3′ end of RNAs, the number and length of U‐tags can provide quantitative information about the binding affinity and regulatory effects of the proteins on their target RNAs. This quantitative aspect enhances the ability to distinguish between different levels of protein–RNA interaction and regulatory impact, providing deeper insights into the functional relevance of these interactions.

RNA Tagging method has been effectively implemented across diverse biological contexts, including yeast and human cells.^[^ ^152^, ^153^, ^154^ ^]^ For instance, applying RNA Tagging to profile the RNA targets of Puf3p, a member of the Pumilio protein family in yeast, revealed its association with numerous mitochondrial mRNAs involved in oxidative phosphorylation and mitochondrial biogenesis. Similarly, studies of the yeast protein Bfr1p using this technique uncovered its extensive interactions with mRNAs linked to endoplasmic reticulum (ER) functions, exposing novel post‐transcriptional regulatory pathways.^[^ ^152^ ^]^ These findings underscore RNA Tagging's utility in mapping RNA–protein interactions and identifying previously unknown regulatory networks.

*APEX‐seq/Chrom‐seq*: As discussed in the RNA‐centric methods, APEX2, which can catalyze the oxidation of biotin‐tyramide by hydrogen peroxide, has been widely used for tagging adjacent proteins.^[^ ^55^, ^155^ ^]^ Recent discoveries have shown that the highly reactive radicals generated by APEX2 can also covalently conjugate to the guanosine in single‐stranded nucleic acids, enabling proximity tagging of RNA.^[^ ^63^, ^156^ ^]^ By leveraging the proximity labeling activity of APEX2, APEX‐seq has been used to study the subcellular localization of RNA. APEX2 is fused to signal peptides directing to various compartments, including the nucleolus, nucleus, endoplasmic reticulum, mitochondria, and cytosol.

Inspired by the success of APEX‐seq, Chrom‐seq has been developed by combining the engineered chromatin mark readers (eCRs) with APEX2 to capture RNAs adjacent to specific epigenetic marks in living cells.^[^ ^157^, ^158^, ^159^ ^]^ By utilizing highly specific eCRs, Chrom‐seq has successfully identified RNA species significantly associated with histone epigenetic modifications such as H3K27me3 (by mCBX7/dPC), H3K9me3 (by mCBX1), and H3K4me3 (by mTAF3), respectively. Chrom‐seq offers significant advantages by eliminating variability and noise associated with antibody quality and crosslinking, and provides a sensitive and efficient way for capturing chromatin‐associated RNAs with fewer living cells. Furthermore, to address the challenge of requiring recombinant expression of the APEX2 fusion protein in the cell of interest, which limiting its applicability to difficult‐to‐transfect cells and tissues, Chrom‐seq protocols compatible with protein‐ or mRNA‐mediated delivery of the engineered chromatin reader‐APEX components are currently under development.^[^ ^157^ ^]^

Although these proximity‐based labeling based methods collectively address limitations of classical techniques, providing scalable, high‐resolution insights into dynamic RNA–protein networks across diverse biological contexts, a notable limitation of these tagging methods is the requirement for the expression of fused enzymes within living cells, including ADAR, APOBEC1, PUP, and APEX2, which restricts their application in primary cells and tissues. Moreover, the expression of these uncontrollably activated enzymes typically requires around 24 hours or more, resulting in high background levels and making it impractical to monitor dynamic RNA binding sites in real time.

## 3 Guidelines for Choice of RNA–Protein Interaction Detection Methods

The methods introduced in this review have catalyzed significant advancements in both basic and translational research, enabling the elucidation of RNA–protein interaction networks across diverse biological contexts.^[^ ^45^, ^101^, ^160^, ^161^, ^162^, ^163^, ^164^ ^]^ When choosing and applying RNA–protein interaction detection approaches, it is important to consider the specific goals of the research, the biological context, and the technical requirements of each method. in vitro methods are usually chosen for studying the fundamental biochemical characteristics of RNA–protein interactions. These methods are simpler, faster, and more cost‐effective, making them ideal for studying direct interactions under controlled conditions, such as temperature, pH, and ion concentrations. Typical in vitro methods include electrophoretic mobility shift assay (EMSA), RNA pulldown, and HTR‐SELEX.^[^ ^165^, ^166^ ^]^ In contrast, in vivo methods are favored when the goal is to understand interactions within the context of living cells or tissues, where RNA–protein interactions may be influenced by the presence of numerous other molecules in cellular environments. When studying RNA–protein interactions in nontransfected host cells, it is advisable to utilize either immunoprecipitation or hybridization‐based methods. In cases where suitable antibody are unavailable, RNA‐centric methods are preferred to determine the RNA–protein interactions. For instance, short RNAs with limited binding sites are well‐suited to methods such as RaPID and RNA‐BioID, which facilitate the expression of the labeling enzymes with target RNA in living cells. Conversely, long and highly expressed RNAs benefit from hybridization‐based techniques like ChIRP and RAP‐MS, owing to their high specificity and efficiency. Although these in vivo methods provide more comprehensive and biologically relevant data, they are also more intricate and time‐consuming, requiring meticulous optimization.^[^ ^167^ ^]^

When deciding between crosslinking and non‐crosslinking approaches for studying RNA–protein interactions, it is important to consider the nature of the interactions you are interested in and the technical requirements of each method. Crosslinking methods involve the use of chemical or UV‐induced crosslinkers to covalently bind RNA to proteins, thereby capturing transient or weak interactions that may otherwise be missed. These methods, such as CLIP‐seq, ChIRP, and RAP‐MS, offer high specificity and efficiency in identifying RNA–protein interactions. However, they can also introduce biases or artifacts due to the crosslinking efficiency itself.^[^ ^168^ ^]^ Non‐crosslinking methods, including proximity labeling‐ and RNA editing‐based approaches, do not rely on crosslinkers but instead on the spatial proximity between RNAs and proteins. These techniques, such as RaPID, CARPID, TRIBE, STAMP, ARTR‐seq, and Chrom‐seq, allow the study of dynamic interaction between RNAs and proteins in living cells. While these methods are generally less disruptive to the natural state of RNA–protein interactions and can be more productive, they may capture candidates that do not interact directly and are more prone to background noise, necessitating stringent controls to improve the signal‐to‐noise ratio. Fortunately, through the development of advanced methodologies, the necessity for crosslinking in RNA‐protein interaction studies has become obsolete. A notable example is the utilization of APOBEC1 within the STAMP to overcome the limitations associated with immunoprecipitation‐based profiling methods by tagging RNA bases in close proximity to a target RBP within living cells. Building upon the enzymatic activity of APOBEC1 observed in vitro,^[^ ^169^ ^]^ the innovative approach of INSCRIBE (IN situ Sensitive Capture of RNA‐protein Interactions in Biological Environments) integrates the strengths of CLIP and STAMP to preserve an authentic map of RBP‐RNA interactions through in situ RNA labeling.^[^ ^170^ ^]^ In the INSCRIBE methodology, the precise localization of the APOBEC1‐nanobody fusion protein to the specific RBP of interest is achieved via nanobody‐primary antibody recognition. By implementing in situ RNA C‐to‐U labeling using the cytosine deaminase APOBEC1 in a streamlined workflow, INSCRIBE enables the identification of transcriptomic RBP–RNA interactions in unaltered cells and primary tissues fixed with either methanol or formaldehyde, without the need for prior plasmid constructions or cellular transformations. Consequently, the integration of STAMP and INSCRIBE represents a significant advancement in enhancing the accuracy of detecting RNA–protein interactions in both live and fixed cellular contexts.

Another critical consideration in RNA–protein interaction studies is whether to use endogenous or exogenous systems. Endogenous system involve studying RNA–protein interactions within their natural cellular context, without expressing RNA or protein of interest. This system ensures that the interactions observed are physiologically relevant and reflective of the natural biological processes. Techniques such as RIP‐seq, CLIP‐seq, LACE‐seq, ARTR‐seq, ChIRP, and CARPID typically use endogenous system to maintain the native cellular environment. However, endogenous methods may pose challenges arising from the unavailability of suitable antibodies or the undetectability of the target RNA or protein within the research models. Exogenous approaches, on the other hand, involve introducing labeled or tagged RNA/proteins into the system to study their interactomes. This can be accomplished using techniques such as RNA pulldown assays or expressing tagged RNA or proteins in cell lines or model organisms. Exogenous methods offer more control over experimental conditions and can facilitate the study of specific interactions with greater ease. But, it is important to note that these methods may not completely replicate the natural context and could potentially introduce artifacts or non‐physiological interactions. Furthermore, with significant advancements in methods for detecting RNA–protein interaction, researchers now have an array of option to consider. For instance, advancements in MS technology are continually improving the sensitivity and accuracy of quantitative protein identification, potentially reducing the amount of starting material required.^[^ ^171^, ^172^ ^]^ Then, a thoughtful selection of the appropriate method will enhance the accuracy and relevance of your findings, finally contributing to a deeper understanding of RNA–protein interactions. Here, we will further share our practical experiences to facilitate the effective exploration on RNA–protein interactions with updated approaches.

**Figure 3:**
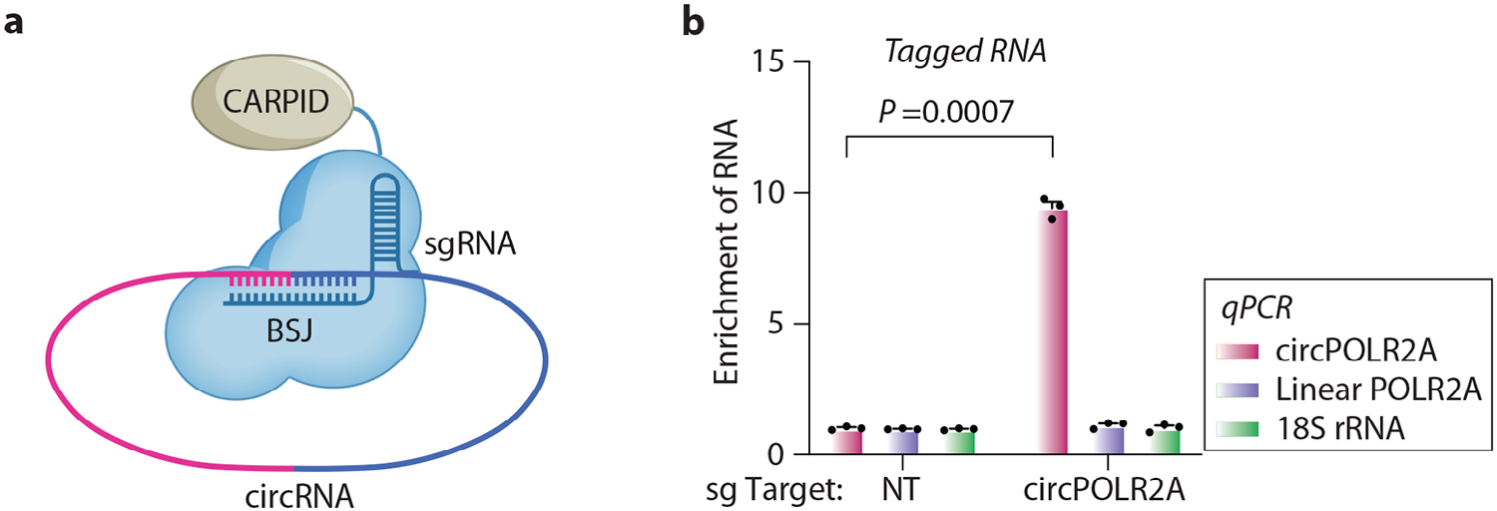
Using CARPID to target circPOLR2A in living HEK293T cells. a) Schematic showing Cas13 targeting of circRNA at the BSJ site. b) CARPID assay followed by qPCR to assess the specificity of circPOLR2A‐targeting sgRNAs in living HEK‐293T cells. Fold enrichment of biotinylated RNA in the circPOLR2A group is normalized against the nontargeting (NT) control. Linear POLR2A and 18S rRNA serve as negative controls. Data are presented as mean ± SD from three independent experiments; statistical analysis was performed using a two‐tailed Student's *t*‐test.

In contrast to proteins, RNA exists in various forms, including rRNA, mRNA, lncRNA, circRNA, small RNA (such as microRNA and small interfering RNA), chromatin‐associated RNA (caRNA), as well as RNA molecules with G‐quadruplex (G4) structures or epigenetic modifications. The diverse nature of RNA species poses substantial challenges in the study of RNA–protein interactions, necessitating advanced methodologies and comprehensive analytical approaches. Specifically, circular ribonucleic acid (or circRNA) is a class of endogenous ncRNAs characterized by a closed‐loop structure, which confers resistance to RNA exonucleases and ensures sustained expression with high stability against degradation.^[^ ^173^ ^]^ CircRNAs are ubiquitous across a wide range of species, from viruses to mammals, and play critical roles in disease initiation and progression through interactions with their molecular partners.^[^ ^174^ ^]^ To date, the interactions between circRNAs and proteins are primarily analyzed using in vitro binding assays or protein‐centric methods, mainly due to the lack of effective approaches to discriminate circRNAs from their cognate linear RNAs.^[^ ^175^, ^176^ ^]^ Inspired by the feasibility of RfxCas13d–BSJ‐gRNA to discriminates circRNAs from linear RNAs, one could apply CARPID or similar technologies to identify the binding proteins of circRNAs.^[^ ^177^ ^]^ Using circPOLR2A as a model, which originates from the POLR2A gene encoding the largest subunit of RNA polymerase II, the investigation into circPOLR2A‐associated proteins involved the execution of in vitro binding and competition screening assays. The results revealed that PKR displayed a distinct preference for interacting with circPOLR2A over its linear counterpart.^[^ ^176^ ^]^ To identify the binding proteins of circPOLR2A, we synthesized a sgRNA that specifically targets the sequence at the back‐splicing junction (BSJ), which is unique to the circRNA and absent in the cognate linear RNA (**Figure** **3**a). To validate the specificity of the circPOLR2A sgRNA with biotinylated RNA, we found only circPOLR2A was significantly enriched, but not the cognate linear POLR2A or 18S rRNA, in the circPOLR2A sgRNA group (Figure 3b). This evidence highlights the efficacy and specificity of the CARPID approach in capturing physiologically relevant protein interactions with circPOLR2A.

## 4 Key Challenges in Studying RNA–Protein Interactions

First, many RNA–protein interactions are transient or characterized by low affinity, posing challenges for conventional methods such as RNA‐SELEX or RNA pulldown. Crosslinking‐based approaches like RIP‐seq and CLIP‐seq can enhance interaction stability but may introduce artifacts or biases. Enzymatic‐based techniques such as RaPID, CARPID, TRIBE, and Chrom‐seq have proven effective in capturing spatial RNA–protein interactions, although they may also involve indirect interactions that require further validation.

Second, there is a pressing need for highly specific and sensitive methods to accurately detect and quantify RNA–protein interactions. Techniques like CLIP‐seq, LACE‐seq, and ARTR‐seq have advanced to offer single‐nucleotide or single‐cell resolution, but they are heavily rely on the availability of high‐quality antibodies. Methods like TRIBE, STAMP, and Chrom‐seq show potential for even finer resolution, they are required the efficiency of ectopic expression of target proteins. Then, it is rational to use suitable methods to determine the interactions between RNA and protein in certain situation.

Third, the advancement of protein detection through MS is a critical component in the study of RNA–protein interactions, particularly at the single‐cell level. While methodologies like STAMP and ARTR‐seq excel in detecting these interactions at single‐cell resolution, RNA‐centric approaches face challenges due to the inability to amplify proteins similar to PCR for oligonucleotides. Efforts to minimize protein or peptide losses during cell isolation, sample preparation, and MS analysis are essential to ensure accurate detection. Significantly, the development of mass spectrometry‐based single‐cell proteomics shows great promise in addressing these challenges,^[^ ^178^, ^179^ ^]^ enabling precise identification of specific biotinylation sites in proteins.^[^ ^180^ ^]^ This advancement has the potential to transform the detection of RNA‐binding proteins at single‐cell resolution with enhanced precision and sensitivity. However, it is worth noting that high‐end mass spectrometry devices can significantly improve protein identification performance but their high cost may still present a barrier to widespread adoption. Continued technological advancements and cost‐reduction strategies may help make these powerful tools more accessible in the future, further advancing research in the field of RNA–protein interactions.

Finally, both proteins and RNA can fold into intricate structures and shift conformations, particularly after undergoing modifications such as phosphorylation for proteins and methylation for RNA. The inherent dynamic nature of these biomolecules poses significant challenges in accurately determining their binding states using conventional biochemical and genomic approaches. Therefore, integrative strategies that combine imaging, biochemical, computational, and other biophysical methods are the most effective in obtaining a comprehensive understanding of RNA–protein interactions. Given the fast development of artificial intelligence (AI) in biomedical research, the integration of computational models would further enhance the interpretation of experimental data, bridging gaps in predicting RBP–RNA interactions.

## 5 Conclusion and Perspective

RNA–protein interactions are central to cellular regulation and disease, necessitating precise and versatile detection methodologies. From hybridization‐based pulldown to CRISPR‐assisted proximity labeling, technological advancements have revolutionized our ability to map RNA–protein interactions with increasing resolution and physiological relevance. These tools have uncovered mechanistic insights into cancer, development, and viral pathogenesis while driving therapeutic innovation.

Despite these progresses, significant challenges remain. Capturing transient or low‐affinity interactions, overcoming cell‐type specificity limitations, and navigating the complexity of high‐throughput datasets demand continual methodological refinement. Future breakthroughs will likely depend on integrating emerging platforms such as CRISPR‐based tools, spatial omics, super‐resolution imaging, and AI to decode the full complexity of the RNA–protein interactome.^[^ ^181^, ^182^, ^183^ ^]^ The convergence of AI and RNA–protein interaction research represents a transformative paradigm shift. With the high‐resolution interaction maps input from conventional experimental techniques like CLIP‐seq, AI‐driven approaches, particularly those leveraging deep learning algorithms and graph‐based models, offer a powerful platform for in silico predication of RBP‐binding sites across transcriptomes with exceptional precision, such as NPI‐GNN,^[^ ^184^ ^]^ ZHMolGraph,^[^ ^181^ ^]^ ProtScan,^[^ ^185^ ^]^ and CoPRA.^[^ ^186^ ^]^ Notably, breakthroughs like AlphaFold in protein structure prediction are expected to enhance our structural understanding of RBPs and their RNA interfaces.^[^ ^187^, ^188^ ^]^ As high‐resolution datasets continue to emerge from advanced techniques like STAMP, ATAR‐seq, and CARPID, the application of self‐supervised and generative AI models hold great promising to unveil hidden regulatory elements, novel binding motifs, and context‐dependent RBPs behaviors.^[^ ^189^ ^]^

Looking ahead, single‐cell sequencing and spatial transcriptomics are set to transform RNA–protein interaction studies. Single‐cell approaches will enable dissection of cellular heterogeneity, capturing rare cell states and transient interactions that are masked in bulk analyses. For instance, coupling methods such as STAMP or SLAM‐RT&Tag with single‐cell RNA‐seq could illuminate how RBP binding dynamics evolve during differentiation or stress responses.^[^ ^129^, ^146^ ^]^ In parallel, spatial transcriptomics also allow the mapping RNA–protein interactions within intact tissue architectures, preserving crucial spatial context for understanding phenomena like tumor microenvironments or neuronal connectivity. Integrating spatial proximity labeling techniques (e.g., APEX‐seq) with multiplexed imaging may reveal subcellular localization governs interaction specificity.^[^ ^63^, ^156^ ^]^

Research on RNA–protein interactions now resides at the intersection of molecular biology, computational science, engineering, and clinical medicine. Interdisciplinary collaboration will be essential to drive this field forward. Computational biologists and AI specialists are instrumental in developing predictive models from multi‐omics datasets, while engineers enhance sequencing and microfluidic systems to boost resolution and throughput. Clinicians, in turn, play a pivotal role in translating these advances into diagnostics tools and therapeutics strategies. Notable examples of such collaborative success include partnerships between CRISPR engineers and RNA biologists, as well as joint efforts from mass spectrometry experts and computational scientists, which have collectively advanced proteomic analysis and led to the development of methods like CARPID.^[^ ^11^ ^]^ Overall, these interdisciplinary advancements collectively set the stage for a new epoch in RNA biology. As these efforts advance, it is promising to unlock novel biomarkers, therapeutic targets, and fundamental biological principles, cementing RNA–protein interaction studies as a cornerstone of molecular biology and precision medicine.

## Conflict of Interest

The authors declare no conflict of interest.

## Peer Review

The peer review history for this article is available in the Supporting Information for this article.

## Supporting information

Supplementary Information: Record of Transparent Peer Review
