# Supplementary material for: Decoding RNA–Protein Interactions: Methodological Advances and Emerging Challenges
Source: Adv Genet (Hoboken). 2025 May 12;6(2):2500011. doi: 10.1002/ggn2.202500011 (PMC12245534; doi:10.1002/ggn2.202500011)
Supplement: Supplementary file 1 — Supplementary Information: Record of Transparent Peer Review [file GGN2-6-2500011-s001.docx]

Record of Transparent Peer Review

Decoding RNA-Protein Interactions: Methodological Advances and Emerging Challenges

Review timeline

Data submitted: 26-Mar-2025

1st Editorial Decision: 06-Apr-2025

Revision Received: 17-Apr-2025

Accepted: 21-Apr-2025

Revision with responses: 17-Apr-2025

***Reviewer #1:***

*This review systematically compares RNA-centric and protein-centric approaches, highlighting their strengths, limitations, and optimal applications. RNA-centric methods, including hybridization-based pulldowns, proximity labeling, and CRISPR-assisted techniques, enable the identification of proteins interacting with specific RNAs, even low-abundance or transient partners. The authors systematically compare techniques, highlight recent innovations (e.g., CRISPR-assisted proximity labeling, LACE-seq, ARTR-seq), and discuss key challenges in the field. The manuscript is well-structured, clearly written, and will serve as a valuable resource for researchers in RNA biology, genomics, and therapeutics. There are some comments as below:*

*Major*

*1. the methodological limitations need deep discussion. The method as CRISPR-assisted methods, Proximity labeling and Single-cell methods was introduced, but the detail is important. As how to control the target binding of dCas13 and RNA. How about the delivery efficiency of RNP complexes, associated with the cell type? maybe authors provide optimization strategies. How do these compare to scRIP-seq or TRIBE at single-cell resolution? A brief discussion would strengthen this section.*

**Response:**

We appreciate the reviewer’s overall positive feedback and constructive advice to further improve the manuscript. Indeed, controlling target binding of dCas13 and RNA is essential for the CRISPR-assisted methods. We have now expanded details about the design of guide RNA, stating that “the binding specificity of dCas13 is intricately linked to the design of gRNAs. Poorly designed gRNAs may result in off-target interactions, particularly in regions of RNA with high structural complexity. Optimization strategies include the selection of target regions with minimal secondary structure, the use of multiple gRNAs to enhance coverage, and the employment of high-fidelity Cas13 variants to mitigate off-target effects.” (page 11, line 357-361).

We also provide more detail about the delivery efficiency of the RNP complexes including that “efficacy of delivering RNP complexes varies depending on the cell type. While lipofection or electroporation can be effective in specific cell lines, primary cells or in vivo systems may necessitate the use of viral delivery systems or nanoparticle formulations. Transient delivery of RNPs is typically preferred to minimize long-term off-target effects, although the efficiency of cellular uptake remains a concern that often requires empirical optimization.” (page 11, line 372-377).

It is important to note that CARPID is an RNA-centric method, while scRIP-seq and TRIBE are both protein-centric methods. Direct comparison between them is difficult. Following the reviewer’s advice, we add a very brief discussion, reading “while many sequencing-based methods could be advanced to study the RBP-RNA interaction in a single-cell resolution, it is still very challenging to develop an RNA-centric method, which mostly depends on MS technique, to identify binding proteins in a single cell. However, the development of MS-based single-cell proteomics shows great progress in addressing these challenges, enabling precise identification of specific biotinylation sites in proteins. This advancement has the potential to transform the detection of RNA-binding proteins at single-cell resolution with enhanced precision and sensitivity.” (page 12, line 391-397).

*2. In part 3, authors provide a guideline for choice of RNA-protein interaction detection methods, and appeared the Figure 3 to verify guideline is suitable. I suggest authors introduce the circPOLR2A interactors from prior studies.*

**Response:**

We appreciate the reviewer’s suggestion. We feel that adding new data without functional verification is inappropriate in this review article and thus we determined to remove it in the revised manuscript but only discuss the potential of studying binding proteins of circular RNAs with the CARPID method. (page 22, line 809-823).

*3. there advantages and limitations will appear all methods, how to select the suitable method is important for research. Authors maybe summary those, this review will more fit or cite.*

**Response:**

We appreciate the reviewer’s insightful advice. We have added more detail about the advantages and limitations of various methods thus to facilitate the selection of suitable methods. We also expand the discussion of how to select different methods in the revised manuscript. (page 7, line 222-230); (page 8, line 242-248); (page 9, line 277-293); (page 11, line 340-361); (page 13, line 472-494); (page 14, line 516-528); (page 15, line 550-557); (page 15, line 571-583); (page 18, line 629-642); (page 18, line 652-665); (page 19, line 680-688); (page 21, line 757-773); (page 22, line 838-852).

*Minor*

*P20 L54-57: AI, Spatial-time transcriptomics and RPI, this will is a top point and perspective. But the reference is poor.*

**Response:**

Thank you. We have now included more references to this part, especially the contribution of AI to RNA-protein interaction prediction. (page 23, line 871-897).

*There is a sugget, all tables maybe integrate a table for veay read and unde stand.*

**Response:**

Thank you. We apologize that we do not fully understand this suggestion.

***Reviewer #2:***

*This review provides an extensive and timely overview of RNA‐protein interaction methodologies, covering both RNA‐centric and protein‐centric approaches. The authors have assembled a comprehensive historical and cutting‐edge techniques, including hybridization‐based pulldowns, proximity labeling, CRISPR‐assisted methods, and RNA tagging strategies. The manuscript is well organized into clear sections that delineate the principles, advantages, limitations, and practical considerations for each method. The inclusion of comparative tables (e.g., Tables 1–6) is commendable as it facilitates direct comparisons and aids researchers in selecting appropriate techniques based on experimental needs. I have a few comments that need to be addressed below:*

*1.	Could the authors also point out the weakness of HyPro-MS method? Although HyPro-MS is a smart design that avoid the genetic engineering. Within intact cells, considering abundant RBP bound to RNA, how can the authors achieve a pefect mapping between the Dig-labelled probe and target RNA molecule?*

**Response:**

We thank the reviewer for his/her overall positive comments and insightful suggestions for improving the manuscript. We agree that it is important to point out the weakness of different methods include HyPro-MS. We have now added the following comments: “To ensure the reliability and accuracy of HyPro-MS, meticulous RNA probe design is critical. One of the primary challenges lies in the complex secondary structures of target RNAs, which can significantly impede hybridization efficiency. Moreover, the choice and optimization of crosslinking methods are crucial, as suboptimal crosslinkers may fail to capture transient or indirect interactions and may introduce non-specific protein associations. Overcoming these limitations requires strategic probe design that accounts for RNA structural features, along with carefully optimized crosslinking protocols to enhance the specificity and sensitivity of protein-RNA interaction detection.” (page 9, line 282-290).

*2.	The sentence “Subsequently, biotinylated oligo(dT)-adapter-B and protein A-Tn5-adapter-A are recruited via a streptavidin-conjugated secondary antibody.” Is not true for RT&Tag. Actually, only the biotinylated oligo(dT)-adapter-B is recruited via a streptavidin-conjugated secondary antibody. In this paragraph, please also discuss the disadvantages of RT&Tag, e.g., it can’t identify the non-polyA transcripts as well as the binding site for RBP*

**Response:**

Thank you very much for the important suggestion. We have now corrected the misleading statement about RT&Tag and also discussed about its limitations. We have revised the sentence to enhance clarity and precision. “Subsequently, biotinylated oligo(dT)-adapter-B is recruited via a streptavidin-conjugated secondary antibody, while protein A-Tn5-adapter-A is concurrently engaged through binding to the same secondary antibody.” (page 15, line 541-543).

Following the reviewer’s suggestion, we have also included the discussion of the disadvantages of RT&Tag, stating that “however, its application is limited in the context of non-polyadenylated RNAs and cytoplasmic RNA analysis”. (page 15, line 559-563).

*3.	There is a typo in the sentence “By leveraing the proximity labelling activity of APEX2, APEX-seq has been employed to study the subcelluar localization of RNA”, leveraing should be leveraging*

*4.	There is a typo in the sentence “Crosslinking methods involve the use of chemical or UV-induced crosslinkers to covalently bond RNA to proteins, thereby capturing transient or weak interactions that may otherwise be missed.”“bond” should be “bind”*

**Response:**

Thank you very much. We have corrected the typos and checked through the manuscript to make sure no more typo exists.

*5.	For both Fig.1 and 2, it is necessary to redefine the contents more explicitly. For example, in Fig.1, the “in vitro” panel is actually for preparation of RNA probes for the downstream application within cells, they are not separated from each other, please indicate the relationship and workflow.*

**Response:**

Thank you very much. We have corrected the inappropriate description of both figures. (figure 1 and 2 were updated).

***Reviewer #3****: This work by Yi et al. is an essential resource for researchers aiming to navigate the evolving landscape of RNA-protein interaction studies and advance their work in RNA biology and precision medicine. Nevertheless, further improvements should be made in the following aspects before final publication.*

*1.It has provided a systematic comparison of multiple methods for studying the RNA-protein interaction, but further elaborations of the comparison of key methods are required. (For instance, when discussing RNA-centric methods, for different hybridization-based RNA pulldown methods (e.g., ChIRP, iDRiP, RAP-MS), their applicability in different biological scenarios could be explored in more detail, in addition to listing their advantages and limitations. For example, for low abundance RNAs, it is more compelling to discuss which method is more effective in identifying proteins interacting with them in what cell types or tissues, taking into account specific cases.)*

**Response:**

We appreciate the reviewer for his/her overall enthusiastic comments and insightful suggestions for strengthening the manuscript. In response, we have expanded the comparison of key RNA-centric hybridization-based methods, including ChIRP, CHART, iDRiP, and RAP-MS, by discussing their specific applicability across different biological contexts. We now highlight their relative strengths in detecting RNA-protein interactions involving low-abundance RNAs and across various cell types and tissues. This revision aims to provide a more nuanced understanding of method selection based on experimental needs and biological scenarios. (page 5, line 151-178); (page 7, line 222-230); (page 8, line 242-248); (page 9, line 277-293); (page 11, line 340-361); (page 11, line 367-377); (page 12, line 391-397).

*2.For protein-centric methods, such as various derivatives of CLIP-seq (e.g. HITS-CLIP, PAR-CLIP, iCLIP, eCLIP), a more profound comparison of their specific differences in terms of data resolution, experimental throughput, and requirements for cell numbers can be made, and specific cases can be provided to illustrate, for which research purpose, which method is more advantageous.*

**Response:**

We appreciate the reviewer’s constructive suggestions. We have now provided a comprehensive description of protein-centric methods, including the data resolution, experimental throughput, and requirements for cell numbers. (page 13, line 472-494); (page 14, line 516-528); (page 15, line 550-557); (page 15, line 571-583); (page 18, line 629-642); (page 18, line 652-665); (page 19, line 680-688); (page 21, line 757-773).

*3.The review could further focus on some emerging technologies that have not yet been widely discussed, such as novel single-cell level-based RNA-protein interaction detection technologies that may have unique advantages in revealing cellular heterogeneity. Additionally, for some methods that combine AI or machine learning, it is more compelling to discuss how they can help predict binding sites for RNA-protein interactions and how these predictions can be used to optimize experimental design and data analysis.*

**Response:**

We are grateful to the reviewer’s insightful advice. We agree that highlighting emerging technologies, particularly those capable of detecting RNA–protein interactions at the single-cell level, would enrich the review by addressing recent advances that are critical for understanding cellular heterogeneity. We have expanded the discussion of AI-integrated methods for predictions in the **Conclusion and Perspective** section. (page 24, line 871-897).

*4.The figures in this review showcase the principles and comparison of RNA-protein interaction approaches, but the design of the figures can be further optimized. In Figure 1 and 2, some illustrations or schematic diagrams can be added to display the operational steps and results of different experiment methods.*

**Response:**

We apologize for the misleading of the figures. We have now revised both figures by adding more details e.g., the critical operational steps. (figure 1 and 2 were updated).

*5.The future direction of RNA-protein interaction research can be discussed in more depth. For example, how will RNA-protein interaction research benefit from the development of emerging technologies such as single-cell sequencing technology and spatial transcriptomics technology? Additionally, RNA-protein interaction research involves several disciplinary fields, hence the importance of interdisciplinary collaboration should be emphasized, and researchers with different disciplinary backgrounds are encouraged to cooperate together on RNA-protein interaction studies.*

**Response:**

Thank you for this thoughtful comment. We have now expanded the discussion of emerging technologies and interdisciplinary collaboration, which will be pivotal in shaping the future of RNA-protein interaction research. (page 24, line 898-911).

Accepted: 21-Apr-2025 with the record of transparent peer review
